# Relevance of Volumetric Parameters Applied to [^68^Ga]Ga-DOTATOC PET/CT in NET Patients Treated with PRRT

**DOI:** 10.3390/diagnostics13040606

**Published:** 2023-02-07

**Authors:** Luca Urso, Angelo Castello, Giorgio Treglia, Stefano Panareo, Alberto Nieri, Ilaria Rambaldi, Matteo Caracciolo, Naima Ortolan, Licia Uccelli, Corrado Cittanti, Massimo Castellani, Mirco Bartolomei

**Affiliations:** 1 Department of Translational Medicine, University of Ferrara, Via Aldo Moro 8, 44124 Ferrara, Italy; 2 Nuclear Medicine Unit, Oncological Medical and Specialist Department, University Hospital of Ferrara, 44124 Cona, Italy; 3 Nuclear Medicine Unit, Fondazione IRCCS Ca’ Granda, Ospedale Maggiore Policlinico, 20122 Milan, Italy; 4 Clinic of Nuclear Medicine, Imaging Institute of Southern Switzerland, Ente Ospedaliero Cantonale, 6501 Bellinzona, Switzerland; 5 Faculty of Biology and Medicine, University of Lausanne, 1011 Lausanne, Switzerland; 6 Faculty of Biomedical Sciences, Università della Svizzera Italiana, 6900 Lugano, Switzerland; 7 Nuclear Medicine Unit, Oncology and Haematology Department, University Hospital of Modena, 41125 Modena, Italy

**Keywords:** volumetric parameters, PRRT, [^68^Ga]Ga-DOTATOC, PET/CT, therapy response assessment, outcomes, survival

## Abstract

Background: this study aims to explore the prognostic and predictive role of volumetric parameters on [^68^Ga]Ga-DOTATOC PET/CT in neuroendocrine tumors (NET) patients treated with peptide receptor radionuclide therapy (PRRT). Methods: We retrospectively evaluated 39 NET patients (21 male, 18 female; mean age 60.7 y) within the FENET-2016 trial (CTiD:NCT04790708). PRRT was proposed with [^177^Lu]Lu-DOTATOC alone or combined with [^90^Y]Y-DOTATOC. [^68^Ga]Ga-DOTATOC PET/CT was performed at baseline and 3 months after PRRT. For each PET/CT, we calculated SUVmax, SUVmean, somatostatin receptor expressing tumor volume (SRETV), and total lesion somatostatin receptor expression (TLSRE), as well as their percentage of changes (Δ), both for liver (_L) and for total tumor burden (_WB). Early clinical response (3 months after PRRT) and PFS were evaluated according to RECIST 1.1 and institutional NET board. Results: Early clinical response identified 9 partial response (PR), 25 stable disease (SD), and 5 progressive disease (PD). Post-SRETV_WB and ΔSRETV_WB were progressively increased among response groups (*p* = 0.02 and *p* = 0.03, respectively). Likewise, median post-SRETV_L was significantly higher in PD patients (*p* = 0.03). SUVmax and TLSRE did not correlate with early clinical response. Median PFS was 31 months. Patients with ΔSRETV_WB lower than −4.17% as well as those with post-SRETV_WB lower than 34.8 cm^3^ showed a longer PFS (*p* = 0.006 and *p* = 0.06, respectively). Finally, multivariate analysis identified ΔSRETV_WB as an independent predictor for PFS. Conclusions: our results could strengthen the importance of evaluating the burden of disease on [^68^Ga]Ga-DOTATOC PET/CT in NET patients treated with PRRT.

## 1. Introduction

Neuroendocrine tumors (NET) are a group of malignancies originating from neuroendocrine cells [1,2]. Despite these types of neoplasms being extremely heterogeneous and occurring from almost every district throughout the body, they usually overexpress somatostatin receptors (SSTR), particularly SSTR-2 [3,4,5]. These receptors are suitable targets for a theranostic approach, with plenty of radiotracers developed, either for single photon emission computed tomography (SPECT), positron emission tomography (PET), or peptide receptor radionuclide therapy (PRRT) [5,6,7,8,9,10]. So far, numerous PET radiopharmaceuticals have been used to study different biological characteristics of these neoplasms. For example, [^68^Ga]Ga-labeled somatostatin analogues ([^68^Ga]Ga-SSTR) and, to a lesser extent, [^64^Cu]Cu-SSTR have been proposed for receptor status evaluation, while fluorine-18 fluorodihydroxyphenylalanine ([^18^F]F-FDOPA) for assessing decarboxylation and storage of amine precursors. Moreover, following recent reports in the literature, [^18^F]F-fluorodeoxyglucose (FDG) PET/CT has progressively gained importance as a prognostic tool in NET diagnostic algorithm [2,11,12]. However, despite the above mentioned evidence, [^18^F]F-FDG PET/CT is currently recommended by official guidelines only for G3 neoplasms, and its best indications and timing are still unsolved and under investigation [13,14,15]. Finally, [^68^Ga]Ga-exendin-4, agonist of the glucagon-like protein-1 receptor, has showed high sensibility and specificity for insulinomas [16,17].

Currently, [^68^Ga]Ga-DOTA-SSTR PET/CT represents the principal diagnostic tool for staging and restaging NET. Indeed, [^68^Ga]Ga-DOTA-SSTR PET/CT must be supported by morphological imaging represented by either computed tomography (CT) or magnetic resonance imaging (MRI) according to the district object of the study. The combined functional and morphologic imaging provides a pooled sensitivity and specificity of 93% and 91%, respectively [15,18,19,20]. When considering patients treated with PRRT, ENETS guidelines [21] suggest treatment evaluation according to response-evaluation criteria in solid tumors (RECIST) 1.1 [22], applied to contrast-enhanced CT, as the main tool for response assessment. However, RECIST 1.1 are questioned in the evaluation of PRRT outcomes due to the possibility of PRRT-induced pseudo-progression or delayed morphological response [14,23,24]. Therefore, further research for new reliable response-evaluation tools is needed.

In this context, a few studies have explored the possible application of semiquantitative parameters extracted from [^68^Ga]Ga-DOTA-SSTR PET/CT in patients treated with PRRT [25,26,27,28]. In particular, volumetric parameters are widely used for interpreting [^18^F]F-FDG PET/CT imaging in several types of malignancies [29,30]. Nevertheless, these parameters were only recently introduced in [^68^Ga]Ga-DOTATOC PET/CT by Abdulrezzak and colleagues [31].

Following these premises, the aim of our study was to explore the prognostic and predictive role of volumetric parameters on [^68^Ga]Ga-DOTATOC PET/CT in a cohort of NET patients treated with PRRT.

## 2. Materials and Methods

This is a pilot study within the prospective phase II clinical trial FENET-2016 (EudraCT: 2016-005129-35—Clinical Trials ID: NCT04790708), currently ongoing at the University Hospital of Ferrara, Italy. The study has been conducted following the approval of the local institutional review board (Protocol N° 160990) and in accordance with the Declaration of Helsinki and the Good Clinical Practice guidelines. Written informed consent was obtained from every patient involved in the study.

### 2.1. Study Population

We retrospectively selected 39 patients (21 males, 18 females, mean age 60.8) treated with PRRT at our Institution from July 2018 to March 2020. Inclusion criteria to be enrolled in this study were: (a) diagnosis of advanced or metastatic NET with confirmed imaging progression, positive [^68^Ga]Ga-DOTATOC PET/CT, and treatment with PRRT within phase II clinical trial FENET-2016; (b) [^68^Ga]Ga-DOTATOC PET/CT available at baseline and at 3 month from the last cycle of PRRT; (c) early clinical-radiological follow-up (3 months after the last cycle of PRRT) performed and available for analysis; and (d) a minimum follow-up of 2 years.

### 2.2. Therapy Protocol

Patients enrolled in the current study were eligible for PRRT according to FENET-2016 inclusion criteria. A therapeutic wash-out for at least 30 days was required, except for cold somatostatin analogues that were withdrawn only for the 14 days preceding radiopeptide infusion. A blood routine sample was performed prior to every therapy administration to evaluate patients’ eligibility for therapy and to exclude toxicity.

PRRT was proposed with two mutually exclusive therapeutic schemes, tailored empirically to each patients’ disease characteristics and extension. The first, the “MONO” scheme, comprehended 5 cycles of 3.7–5.55 GBq of [^177^Lu]Lu-DOTATOC, with a cumulate activity of 18.5–27.75 GBq (500–750 mCi); the second, the “DUO” scheme, comprehended 3 cycles of 3.7–5.55 GBq of [^177^Lu]Lu-DOTATOC alternating with 2 cycles of 1.85–2.775 GBq of [^90^Y]Y-DOTATOC, with a cumulate activity of 11.1–16.65 GBq of [^177^Lu]Lu-DOTATOC and 3.7–5.55 GBq of [^90^Y]Y-DOTATOC. An interval of 8–10 weeks was observed between every cycle. Main criteria guiding the choice of the therapy scheme were already described in a previously published paper from our group [14].

### 2.3. Images Acquisition and Evaluation

Images were acquired from the mid-thigh to the skull vertex 50–70 min after [^68^Ga]Ga-DOTATOC injection (150 ± 50 MBq) using a standard technique on a dedicated PET/CT system (Biograph mCT Flow; Siemens Medical Solutions, Malvern, PA, USA). After noncontrast-enhanced low-dose CT (120 keV, 80 mAs, CareDose; reconstructed with a soft-tissue kernel to a slice thickness of 3 mm), PET was acquired in 3-dimensional mode (matrix, 200 × 200) using FlowMotion system. The emission data was corrected for randoms, scatter, and decay. Attenuation correction was performed using the nonenhanced low-dose CT data.

**Figure 1:**
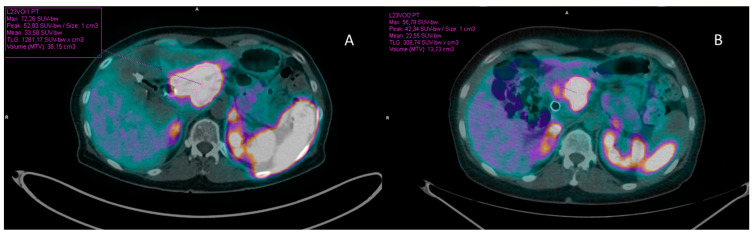
Illustrates the procedure to semiautomatically calculate the metabolic parameters, including those objects of the study, such as SRETV and TLSRE in baseline (**A**) and post-PRRT [^68^Ga]Ga-DOTATOC PET/CT in a patient affected by a pancreatic NET (**B**).

All images were processed and analyzed on a Syngo.via workstation (Siemens Healthineers, Enlargen, Germany) by two experienced nuclear medicine physicians. For SUVmax and SUVmean calculation, circular regions of interest (ROI) were drawn in transaxial slices around the tumor lesions with focally increased uptake. Every ROI was automatically adapted by the software into a 3-dimensional volume of interest (VOI) (Figure 1). In addition to SUV measurements, two more volumetric parameters, namely, somatostatin receptor expressing tumor volume (SRETV) and total lesion somatostatin receptor expression (TLSRE) were calculated for each [^68^Ga]Ga-DOTATOC PET/CT, in accordance with previous studies [31,32]. SRETV, analogue to [^18^F]F-FDG metabolic tumor volume (MTV), represents the tumor volume with at least 40% uptake of SUVmax within the VOI. TLSRE, corresponding to [^18^F]F-FDG total lesion glycolysis (TLG), was calculated by multiplying SUVmean and SRETV within the same VOI. The sum of the volumetric parameters of each hepatic lesion was used to obtain liver tumor burden (SRETV_L and TLSRE_L). The sum of the 5 most relevant lesions according to RECIST 1.1 criteria [22] was used to define total tumor burden (SRETV_WB and TLSRE_WB). All PET/CT parameters were calculated both at baseline (pre-) and at first restaging (post-) [^68^Ga]Ga-DOTATOC PET/CT. Moreover, their variations were calculated as well, and herein indicated as delta (∆).

### 2.4. Early Clinical Response Evaluation and Follow-Up

In consistence with FENET-2016 protocol, PRRT response assessment was multidisciplinarily discussed at the institutional tumor board according to the patients’ clinical conditions and radiological response and performed 3 months after the last cycle of PRRT. Radiological evaluation was performed following RECIST 1.1 criteria [22]. Patients were consequently divided into the following groups of treatment response: Complete Response (CR), Partial Response (PR), Stable Disease (SD), and Progressive Disease (PD). Likewise, disease progression at follow-up was considered in case of radiological and/or clinical evidence of progression.

### 2.5. Statistical Analysis

Descriptive statistics were performed using conventional metrics (mean, median, range). Differences in the various clinical and metabolic parameters were computed using Fisher’s exact, Mann–Whitney test, and Kruskal–Wallis test as appropriate. Progression-free survival (PFS) was calculated as the duration between the date of the first cycle of PRRT and that of either disease progression or death. PFS was analyzed using the Kaplan–Meier method and the log-rank test. A Cox proportional-hazards regression analysis was used to evaluate factors independently associated with PFS. All statistical analyses were carried out using the Statistical Package for Social Sciences, version 23.0, for Windows (SPSS, Chicago, IL, USA), and *p* values < 0.05 were considered to be statistically significant.

## 3. Results

### 3.1. Patients’ Characteristics

Twenty-three patients (59%) were treated with MONO scheme with a mean cumulative activity of 24.2 ± 2.7 GBq, whereas the other sixteen patients (41%) received DUO scheme with a mean cumulative activity of 20 ± 1.5 GBq.

Nineteen patients (48.7%) were affected by NET originating from pancreas, seven (17.9%) from midgut, six (15.4%) of bronchial origin, in five patients (12.8%) the primary origin was unknown, while one patient (2.6%) had paraganglioma and cerebral primary origin. Histological examination demonstrated a G1 NET in 4 patients, G2 in 31, and G3 in 3 patients, whereas for 1 patient, grading was not available. The proliferation index, expressed by median Ki-67, was 8% (range 1–35%). Patients’ features are reported in Table 1.

Among the clinical variables, median SRETV_TB and TLSRE_TB at baseline were significantly higher in NET of unknown primary origin (*p* = 0.025, *p* = 0.031, respectively). Similarly, median SRETV_L and TLSRE_L, both at baseline and at the first evaluation, were significantly higher in patients affected by NET of unknown primary origin (*p* = 0.019, 0.025, 0.015, and 0.02 respectively).

No other significant differences were observed according to age, sex, functioning vs. nonfunctioning tumors, and therapy scheme (MONO vs. DUO). No correlation was found between volumetric parameters and Ki67 as well.

### 3.2. Volumetric Parameters and Early Clinical Response

No patient had a CR to treatment, whereas PR occurred in 9 patients (23.1%), SD in 25 patients (64.1%), and PD in 5 patients (12.8%). Overall, PRRT responders (PR and SD patients) were 34 (87.2%).

#### 3.2.1. Total Tumor Burden Analysis

**Figure 2:**
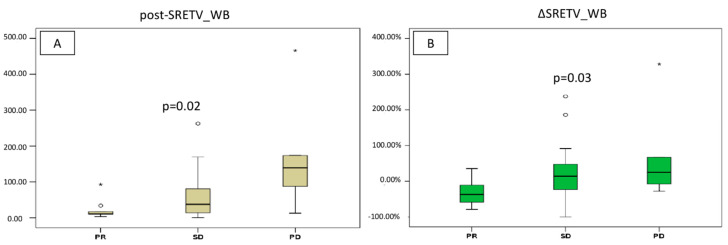
Graphical box-plots representing median values of somatostatin receptor expressing tumor volume (SRETV) for total tumor burden (_WB) at first restaging (**A**) and according to variation between baseline and restaging (Δ) (**B**). Both median values were significantly different among response categories. Outliers and extremes are represented by ° and *, respectively.

According to our results, post-SRETV_WB increased progressively from PR to PD, and median value results were statistically different among the three groups: 11.3 (range 3.2–92.7) for PR, 37.4 (range 0.4–821.1) for SD, and 139.2 (range 13–465.7) for PD (*p* = 0.02). Likewise, when comparing percentage change of SRETV between baseline and at first restaging (i.e., ΔSRETV_WB), the reduction was significantly higher in patients with PR. Of note, median ΔSRETV_WB was −37.4% (range −78–8% +35.2%) for PR, +14.5% (range −99.7–237.5%) for SD, and +24.8% (range −27.9–328.4%) for PD (*p* = 0.03) (Figure 2A,B).

#### 3.2.2. Liver Tumor Burden Analysis

**Figure 3:**
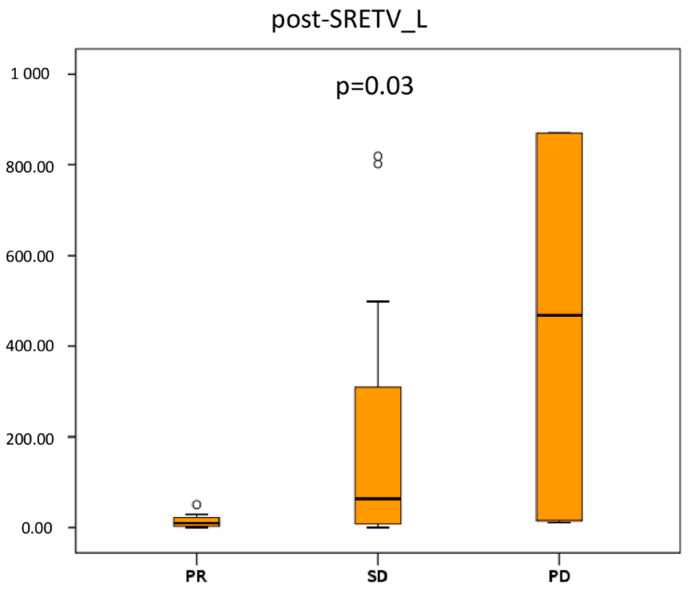
Graphical box-plots representing median values of somatostatin receptor expressing tumor volume (SRETV) for liver tumor burden (_L) at first restaging. Liver tumor burden was significantly greater in patients with progressive disease (PD). Outliers are represented by °.

Median post-SRETV_L was significantly different in the three response groups: 10.1 (range 0–50.7) for PR, 63.8 (range 0–1320.6) for SD, and 468.4 (range 11.4–2272) for PD (*p* = 0.03) (Figure 3). On the other hand, the other metabolic parameters (i.e., SUVmax, SUVmean, and TLSRE) were not associated with clinical-radiological response, both for total and liver tumor burden.

### 3.3. Volumetric Parameters and Clinical Outcomes

**Figure 4:**
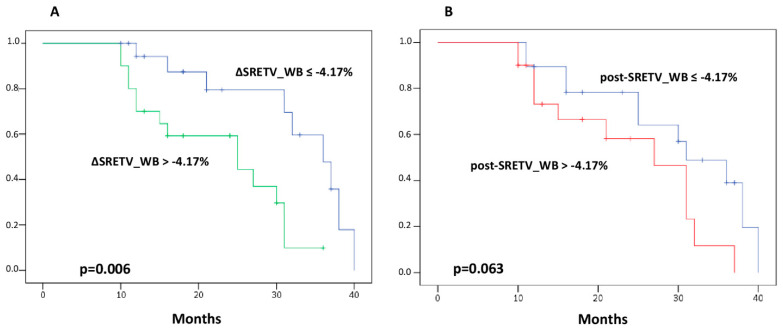
Progression-free survival curves with log rank test obtained for somatostatin receptor expressing tumor volume (SRETV) of total tumor burden (_WB) as percentage change (**A**) and at first restaging (**B**).

For the entire group of patients included in the analysis, median PFS was 31 months (95% CI 26.8–35.2). According to the Kaplan–Meier curve, patients with ΔSRETV_WB below the median value had a longer PFS than those with values above −4.17% (median PFS, 36 vs. 25 months, respectively; *p* = 0.006) (Figure 4A). Similarly, patients with median post-SRETV_WB greater than median value (i.e., 34.8) had a tendency toward a shorter PFS than those with post-SRETV_WB below median value (median PFS, 27 vs. 31 months, respectively; *p* = 0.063) (Figure 4B). On the other hand, no significant association between PFS and SRETV_L was found, as well as for PFS and all TLSRE parameters, both for total tumor burden and for liver tumor burden.

Upon univariate analysis, median ΔSRETV_WB ≤ −4.17% at early evaluation was significantly associated with longer PFS (HR 0.275, 95% CI 0.102–0.744; *p* = 0.011). On the contrary, for the univariate analysis for PFS, no significant differences were found with respect to patient age, gender, grading, type of PRRT scheme, site of primary tumor, and other PET/CT parameters derived from both total body and liver tumor burden, while Ki-67 and median SRETV at first restaging showed only a tendency for PFS (*p* = 0.064 and 0.078, respectively). Finally, multivariate analysis, including all variables with *p* < 0.09 for the univariate analysis, showed that median ΔSRETV_WB ≤ −4.17% was independently associated with PFS (HR 0.296, 95% CI 0.105–0.832; *p* = 0.021).

The results of univariate and multivariate analyses are summarized in Table 2.

## 4. Discussion

In the last ten years, PRRT has become a consistent therapeutic option for advanced, metastatic NET overexpressing SSTR at PET imaging [8]. In our cohort of patients, PRRT was confirmed as an effective treatment, reaching a disease control rate of 87.2%, consistent with prior reports in the literature [33]. However, an intense overexpression of SSTR at baseline imaging is not always enough for reaching disease control after PRRT. Besides the NETest, which represents a bright opportunity for the future early identification of PRRT nonresponders, some more reliable parameters are needed to identify patients who can most likely benefit from PRRT, as well as for assessing response to therapy [34]. Recently, volumetric parameters extracted from [^68^Ga]Ga-DOTA-SSTR PET/CT have been utilized in several research papers with promising results in terms of outcome predictions in various settings of disease [32,35,36,37]. The aim of our work was to assess the relevance of volumetric parameters extracted by [^68^Ga]Ga-DOTATOC PET/CT to predict early clinical response and long-term outcomes of NET patients treated with PRRT. We found that both median post-SRETV_WB and post-SRETV_L were capable of significantly discriminating early clinical response groups. Ezzidin et al. [38] already reported a correlation between baseline liver tumor burden ≥25% on radiological imaging and OS after PRRT. Furthermore, Durmo et al. [26] recently discriminated PRRT-responders from nonresponders according to baseline SRETV (which they called bTV), similarly to the work previously published by Pauwels et al. [25]. To the best of our knowledge, this is the first work reporting that post-SRETV_WB and post-SRETV_L on [^68^Ga]Ga-DOTATOC PET/CT both correlated with early clinical response. This finding highlights that [^68^Ga]Ga-DOTATOC PET/CT could also be important in the assessment of response to PRRT, which is actually performed only with RECIST 1.1 criteria on ceCT. Moreover, our study strengthens the results of Durmo and colleagues [26], highlighting the importance of a volumetric evaluation of the disease burden in patients treated with PRRT, both at baseline and at follow-up [^68^Ga]Ga-DOTATOC PET/CT.

Most meaningfully, we found a statistically significant correlation between median ΔSRETV_WB and early clinical response to PRRT. Moreover, the same parameter was associated with longer PFS after PRRT (median value −4.17%) and identified as an independent predictor for PFS by multivariate analysis. This result was not unexpected, as Toriihara et al. [32] also reported a correlation between the whole body SRETV on [^68^Ga]Ga-DOTATATE PET/CT and PFS in 92 NET patients. However, considering only patients treated with PRRT, we did not find any similar result in the literature. This finding, together with the abovementioned one, could strengthen the importance of [^68^Ga]Ga-DOTATOC PET/CT in the response evaluation after PRRT if confirmed in a larger cohort of patients. Indeed, response assessment still represents a challenge for clinicians, as RECIST 1.1 criteria, suggested as the standard of reference by current guidelines, are known to be frequently impaired by late response, or necrosis-induced pseudo-progression [23]. Volumetric parameters, extracted from [^68^Ga]Ga-DOTATOC PET/CT, could cope with this issue, providing a more accurate evaluation of patients’ early response to PRRT and helping clinicians in predicting patients’ outcomes. In particular, patients with a high tumor volume burden of disease on [^68^Ga]Ga-DOTATOC PET/CT after PRRT may need a closer and tighter follow-up, as these patients seem more prone to disease progression in a shorter period of time.

Conversely, none of the remaining parameters extracted from [^68^Ga]Ga-DOTATOC PET/CT showed any correlation with either early clinical response to PRRT, or PFS. ΔSUVmax on [^68^Ga]Ga-DOTATOC PET/CT has already been labeled as unreliable in the assessment of response to PRRT [39]. Indeed, SUVmax describes only the hottest voxel within a contoured ROI and does not necessarily represent the receptor status of a whole lesion, let alone the whole burden of disease. This is especially true for NET lesions that are often heterogeneous due to polyclonal disease [40,41]. For these reasons, ΔSUVmean could be, theoretically, more accurate than ΔSUVmax in the evaluation of therapy response. However, larger lesions, especially those with a necrotic core, could be less reliably evaluable with SUVmean. As a consequence, TLSRE, which is derived by SUVmean, can reflect the same problems. Conversely, SRETV may be more accurate in distinguishing the real load of disease, because it is not affected by the nonviable part of NET lesions, which are excluded from the set threshold.

This study presents a few limitations. First, the sample size analyzed was quite small; thus, we encourage future larger studies. Second, patients included in the study were heterogeneous, as NET of various primary origin and clinical stage were included. It would be interesting to evaluate the different parameters extracted from [^68^Ga]Ga-DOTATOC PET/CT in a larger cohort of patients with a common primary origin. Moreover, patients had received different kinds of treatments before PRRT, which may represent another source of heterogeneity. Finally, taking RECIST 1.1 as a reference, we have only considered the sum of the 5 most relevant lesions for assessing treatment response. Nevertheless, this method may not reflect completely tumor extension. Hopefully, the evolution of new semiautomatic software will provide us with more accurate and less time-consuming systems, which may allow to consider each single lesion and better define the total disease burden.

## 5. Conclusions

Volumetric parameters extracted from [^68^Ga]Ga-DOTATOC PET/CT performed 3 months after the last cycle of PRRT (i.e., ΔSRETV_WB, post-SRETV_WB, and post-SRETV_L) were associated with early response to PRRT. Moreover, ΔSRETV_WB was found to be a prognostic and predictive factor for PFS in NET patients treated with PRRT. An increase of total tumor burden might indicate a higher risk for progressive disease after PRRT. It might also justify a closer follow-up and an early change of therapeutic strategy. Therefore, our preliminary results, if confirmed in a larger cohort of patients, could encourage the use of volumetric parameters extracted from [^68^Ga]Ga-DOTATOC PET/CT for therapy response assessment of NET patients treated with PRRT.

## Figures and Tables

**Table 1:** Patients’ features.

Patients	Feature	N (%)	
Gender, *n*(%)	Male	21 (53.8)	
	Female	18 (46.2)	
Age, median	60.7 [25–80 y]		
Primary origin	Midgut	7 (17.9)	
	Pancreatic	19 (48.7)	
	Cerebral	1 (2.6)	
	Bronchial	6 (15.4)	
	CUP	5 (12.8)	
	Pheocromocytoma	1 (2.6)	
Grading (%)	Median 8% [1–40%]	G1	4 (19.3)
		G2	31 (77.4)
		G3	3 (3.3)
NET Syndrome	Functioning	9 (23.1)	
	Nonfunctioning	30 (76.9)	
PRRT Scheme	MONO	23 (59)	
	DUO	16 (41)	

Abbreviations: CUP = NET of unknown primary origin; N = number; NET = neuroendocrine tumors; PRRT = peptide receptor radionuclide therapy.

**Table 2:** Univariate and multivariate survival analysis.

		Univariate Analysis			Multivariate Analysis		
		HR	95% CI	*p* Value	HR	95% CI	*p* Value
**Progression-free survival (PFS)**							
Age	per year	0.981	0.945–1.018	0.310			
Gender	Male vs. Female	1.056	0.448–2.490	0.901			
Primary origin	Pancreas vs. others	1.726	0.722–4.124	0.219			
NET syndrome	Functioning vs. nonfunctioning	1.147	0.657–2.001	0.630			
Median Ki-67	<8 vs. ≥8	2.418	1.051–6.148	0.064	0.223	0.192–1.468	0.223
Grading	G1 vs. G2 vs. G3	3.020	0.289–31.51	0.356			
		0.378	0.060–2.387	0.301			
		0.931	0.266–3.266	0.911			
PRRT scheme	Mono vs. Duo	0.962	0.630–1.470	0.858			
post-SRETV_WB	≤34.8 vs. >34.8	0.452	0.187–1.092	0.078	0.422	0.164–1.092	0.075
post-SRETV_L	≤35.1 vs. >35.1	0.680	0.284–1.632	0.389			
ΔSRETV_WB	≤−4.17 vs. >−4.17	0.275	0.102–0.744	**0.0** **11**	0.295	0.107–0.817	**0.019**
ΔSRETV_L	≤20.1 vs. >20.1	0.770	0.324–1.830	0.554			
post-TLSRE_WB	≤763.3 vs. >763.3	0.519	0.235–1.722	0.155			
post-TLSRE_L	≤585.2 vs. >585.2	0.680	0.284–1.632	0.389			
ΔTLSRE_WB	≤−2.38 vs. >−2.38	0.489	0.205–1.166	0.107			
ΔTLSRE_L	≤12.6 vs. >12.6	0.489	0.205–1.166	0.630			

Abbreviations. Δ: variation between baseline and restaging; _L: liver; _WB: whole-body; PRRT: peptide receptor radionuclide therapy; SRETV: somatostatin receptor expressing tumor volume; TLSRE: total lesion somatostatin receptor expression.

## Data Availability

The data presented in this study are available upon motivated request to the corresponding author.
